# Preliminary molecular genetic analysis of the Receptor Interacting Protein 140 (RIP140) in women affected by endometriosis

**DOI:** 10.1186/1743-1050-2-11

**Published:** 2005-08-30

**Authors:** Virginia Caballero, Rocío Ruiz, José Antonio Sainz, Marina Cruz, Miguel Angel López-Nevot, José Jorge Galán, Luis Miguel Real, Francisco de Castro, Vicente López-Villaverde, Agustín Ruiz

**Affiliations:** 1 Department of Structural Genomics. neoCodex. Averroes N°8. Edf. Acrópolis 110-1. 41020 Seville, Spain; 2 Unidad de Reproducción. Servicio de Obstetricia y Ginecología. Hospital de Valme, Ctra. Cádiz, s/n 41014 Seville, Spain; 3 Servicio de Análisis Clínicos. Hospital Universitario Virgen de las Nieves. Avda. Fuerzas Armadas, 2 18014 Granada, Spain; 4 Unidad de Reproducción Humana Asistida. Hospital Universitario Príncipe de Asturias. Ctra. Alcalá-Meco s/n. 28805 Madrid. Spain

## Abstract

**Background:**

Endometriosis is a complex disease affecting 10–15% of women at reproductive age. Very few genes are known to be altered in this pathology. RIP140 protein is an important cofactor of oestrogen receptor and many other nuclear receptors. Targeting disruption experiments of *nrip1 *gene in mice have demonstrated that nuclear receptor interacting protein 1 gene (*nrip1*), the gene encoding for rip140 protein, is essential for female fertility. Specifically, mice null for *nrip1 *gene are viable, but females are infertile because of complete failure of mature follicles to release oocytes at ovulation stage. The ovarian phenotype observed in mice devoid of rip140 closely resembles the luteinized unruptured follicle (LUF) syndrome that is observed in a high proportion of women affected of endometriosis or idiopathic infertility. Here we present a preliminary work that analyses the role of *NRIP1 *gene in humans.

**Methods:**

We have sequenced the complete coding region of *NRIP1 *gene in 20 unrelated patients affected by endometriosis. We have performed genetic association studies by using the DNA variants identified during the sequencing process.

**Results:**

We identified six DNA variants within the coding sequence of *NRIP1 *gene, and five of them generated amino acid changes in the protein. We observed that three of twenty sequenced patients have specific combinations of amino-acid variants within the RIP140 protein that are poorly represented in the control population (p = 0.006). Moreover, we found that Arg448Gly, a common polymorphism located within *NRIP1 *gene, is associated with endometriosis in a case-control study (59 cases and 141 controls, p_allele positivity test _= 0.027).

**Conclusion:**

Our results suggest that *NRIP1 *gene variants, separately or in combinations, might act as predisposing factors for human endometriosis.

## Background

Endometriosis (Online Mendelian Inheritance in Man (OMIM) 131200) is a complex disease affecting to 10–15% of women at reproductive age. The disease consists in pelvic pain and infertility due to the existence of endometrial glands and stroma outside the uterine cavity [1]. Anovulatory cycles and Luteinized Unruptured Follicle syndrome (LUF) are also evident in a great proportion of affected women [2,3]. Moreover, recurrent pregnancy losses, low quality of oocytes and early embryo loss in women with endometriosis have been suggested [4,5].

Receptor Interacting Protein 140 (RIP140) (Swiss-Prot P48552) is a high pleiotropic protein that acts as a co-regulator of multiple members of the nuclear receptor super-family including oestrogen, progesterone, retinoid acid or glucocorticoid receptors. Targeting disruption experiments of this function in mice have demonstrated that *nuclear receptor interacting protein 1 *(*nrip1*) gene (GenBank NM_173440), the gene encoding for rip140 protein, is essential for female fertility [3]. Specifically, mice null for *nrip1 *gene are viable, but females are infertile because of the complete failure of mature follicles to release oocytes at ovulation stage [3]. The ovarian phenotype observed in mice devoid of rip140 closely resembles the LUF syndrome that is observed in a high proportion of women affected by endometriosis or idiopathic infertility [2,3]. In addition, embryo transfer and ovarian transplantation experiments in *nrip1 *knock-out mice indicate slightly longer pregnancies in *nrip1*-/- mice and a high rate of foetal and neonatal losses of pups from mothers with *nrip1*-/- ovaries [6]. These data suggest that rip140 protein may have two functions in mice ovaries: i) an essential role in ovulation; ii) a secondary role in the maintenance of pregnancy [3,6]. More recently, a role for *nrip1 *gene in fat accumulation has been also proposed [6].

Due to *nrip1*-/-, female mice have several traits that closely resemble endometriosis findings; we decided to explore the role of the human *NRIP1 *gene (GenBank NM_003489) in women affected by endometriosis. Direct molecular analysis of endometriotic tissue specimens revealed no de novo mutations in 20 affected tissues. However, different germ-line genetic variants have been detected during our study. The involvement of these germline variants with endometriosis is proposed.

## Methods

### Patients

Endometriosis was defined according to the endometriosis classification system of the American Society for Reproductive Medicine (1996) [7]. All patients included correspond to stage III or IV of endometriosis. The initial sequencing project analyzed the complete sequence of *NRIP1 *gene in 20 independent DNA samples obtained from fresh endometriotic tissue derived from peritoneal implants or endometrioma lesions of 20 unrelated women with severe endometriosis. We also obtained fresh blood samples of these patients to test the germ-line or the somatic nature of the DNA variants detected.

To perform association studies between *NRIP1 *gene and human endometriosis, we genotype three groups of individuals. i) We increase the sample size of the case group (endometriosis group) three-fold using germ-line DNA derived from blood of 39 additional women affected by severe endometriosis (Stage III-IV). Therefore, the sample size of the endometriosis group for the association studies conducted was 59 (118 chromosomes). ii) To estimate population frequencies of mutations or polymorphism detected, 94 unselected and unrelated controls from the same geographical region were genotyped in an anonymous fashion (188 chromosomes). iii) A "super-control" group consisted of 47 healthy and fertile women without any sign or symptom of endometriosis, normal response to gonadotrophins and conserved ovulation (94 chromosomes) was also studied.

The ethnicity background of all probands and controls was Caucasian (white Europid) minimizing the possibility of population stratification in our case-control studies. The referral centers for this study are the Hospital de Valme (Seville), the Hospital Universitario Virgen de las Nieves (Granada), and the Hospital Príncipe de Asturias (Alcalá de Henares, Madrid). Informed consent was obtained from all patients. The institutional review board of referral centers has approved our research.

### DNA extraction

We obtained 5 ml of peripheral blood from all patients to isolate germline DNA from leukocytes and about 100 μg of fresh endometriotic tissue during a programmed laparoscopic intervention of the patients. DNA extraction was performed according to standard procedures using Nucleospin Blood Kit (Macherey-Nagel) or alternative protocols. To perform Polymerase Chain Reactions (PCRs), we prepared aliquots of DNA at a concentration of 5 ng/μl. The rest of the stock was cryopreserved at -20°C.

### Mutation analysis

*NRIP1 *cDNA was first cloned by Cavailles et al. [8]. This gene is mono-exonic, spans 7,239 base pairs (bp) and is located at 21q11.2. Genomic sequence containing *NRIP1 *gene was identified using the blat tool at UCSC Genome Bioinformatics server . Information concerning any Single Nucleotide Polymorphism (SNP) or mutation identified was compared with the UCSC Genome Bioinformatics server and also with the Single Nucleotide Polymorphism Database (dbSNP) at the National Centre for Biotechnology Information (NCBI) . According to standard mutation nomenclature [9], we employed the most frequent allele in the first position and the rarer allele in the last position.

We employed automated DNA sequencing methods to scan the entire coding sequence of *NRIP1 *gene in selected specimens. Overlapping PCRs covering the entire gene were designed and PCR products were purified and bi-directionally sequenced using the corresponding pair of primers (Table 1). Sequencing reactions were performed using the CEQ Dye Terminator Cycle Sequencing Quick Start Kit (Beckman Coulter, Inc) according to the manufacture's instructions. Fluorograms were analyzed on CEQ™ 8000 Genetic Analysis System following the manufacturer's instructions (Beckman Coulter, Inc).

**Table 1:** Amplification primer sequences and PCR product size. *NRIP1 *is a monoexonic gene. We designed eight overlapping amplicons to cover the entire coding sequence of this gene.

**PRIMER**	**SEQUENCE 5' → 3'**	**PCR product size (bp)**
1F	TTCTAGTTCTGCCTCCTTAAC	554
1R	ACATTTCTGGCAGTGCATTTC	
2F	GATCAGGTACTGCCGTTGA	528
2R	CGAATCTTCCTGATGTGACT	
3F	GTGCTATGGTGTTGCATCAAG	572
3R	TGCAGGTTATAAGAACTCACTGG	
4F	CATCATCAAGCAAACTGATGGC	577
4R	AGCCCTCAGGGAGTACACAA	
5F	CTTCAATTGCTACTTGGCCAT	582
5R	GTAGTCAACCAACAGGTCCT	
6F	CTGGAAACACAGATAAACCGATAGG	584
6R	TGGCACTTCTAGAATCAAAG	
7F	AGATAGTTACCTGGCAGATG	572
7R	TCCTACTTTCCCTGAGCACT	
8F	CAGTTGCATGGATAACAGGA	645
8R	GTATTGGTTACTGGTGATG	

### Genotyping

To verify the DNA variants detected during the sequencing process and to perform association studies, we employed Fluorescent Resonance Energy Transfer (FRET) protocols. We designed and synthesized amplification primers and fluorescent detection probes for all the DNA variants identified within the *NRIP1 *gene. The selected primer pairs and detection probes are summarized in Tables 1 and 2. Real-time PCR was performed in the LightCycler system (Roche Applied Science) using reaction conditions previously published by us [10].

**Table 2:** Anchor and Sensor probes sequences employed for coding Single Nucleotide Polymorphism (cSNP) analysis using Fluorescence Resonance Energy Transfer (FRET) technology.

**Mutation**	**PROBE**	**SEQUENCE 5' → 3**
Gly75Gly	75-ANCHOR	AGTAATGGTCCAGTTCTCAATACAC – F
	75-SENSOR	Cy5 – TACATATCAGGGGTCTGGC – Ph
His221Arg	221-ANCHOR	Cy5 – AGTGGAACAAAGGTCATGAGTGAAC – Ph
	221-SENSOR	TCTCCTCATCATGTTGGACA – F
Ile441Val	448-ANCHOR	TATTCCAACTGTGTTCCCATAGACT – F
Arg448Gly	448-SENSOR	Cy5 – GTCTTGCAAACACCGAACTG – Ph
Ser803Leu	803-ANCHOR	GCGCACCTGCCTTACCAGTGTCCCGA – F
	803-SENSOR	Cy5-GACTTTAAATCGGAGCCTGTT – Ph
Val1079Phe	1079-ANCHOR	Cy5 – CGAGAAACACAAGACAAGGACATTT – Ph
	1079-SENSOR	GGAGGCAATTCTGTTACCAG – F

Nomenclature: F: Fluoresceine, Ph: Phosphate.

**Figure 1:**
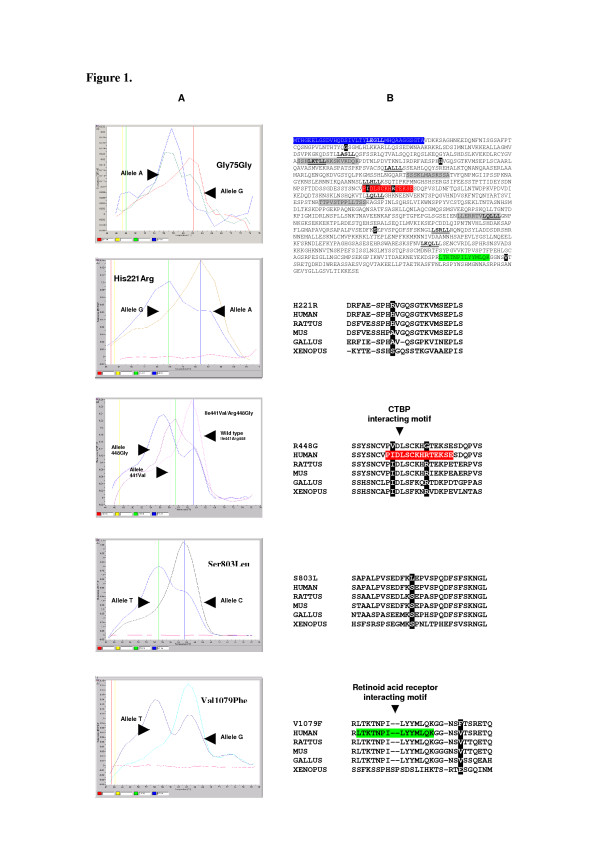
Detection of germ line variants in *NRIP1 *in patients with severe endometriosis. A) Spectrofluorimetric analysis of *NRIP1 *gene using real-time PCR. Analysis of the fluorescence measured during melting curve determination in the LightCycler (Roche Applied Science). Each allele has a specific melting point and all alleles are represented by its specific nucleotide change with the exception of Ile441Val and Arg448Gly polymorphisms. Nt c.512 G->A (Gly75Gly, melting points, Allele G: 62°C; Allele A: 57°C). Nt c.949 A->G (His221Arg, melting points, Allele A 61°C; Allele G: 56°C). Nt c.1608 A->G (Ile441Val) and Nt c.1629 C->G (Arg448Gly) (melting points, Allele Val441: 59°C; Allele Gly448: 56°C; wild type: 63°C). Nt c.2695 C->T (Ser803Leu, melting points, Allele C: 61°C; Allele T: 55°C). Nt c.3522 G->T (Val1079Phe, melting points, Allele G: 62°C; Allele T: 54°C). B) Sequence conservation and location of mutations in the RIP-140 protein. Black shading indicates the position of mutations. LXXLL motifs responsible for ligand independent interaction with Retinoid Acid Receptor (RAR) and Retinoid X Receptor (RXR) are in bold and underlyned. Signal peptide is depicted in blue, Carboxyl terminal binding protein (CTBP) and RAR interacting motifs are in red and green respectively. Low complexity regions are shown in grey.

The conditions to obtain optimal melting curves for FRET analysis and spectrofluorimetric genotypes were 95°C for 0 s, 63°C for 25 s, 45°C for 0 s and 80°C for 0 s (with a temperature-transfer speed of 20°C/s in each step, except the last step, in which the speed of temperature transfer was 0.1°C/s). In the last step, a continuous fluorometric register was performed fixing the gains of the system at 1, 50, and 50 on channels F1, F2, and F3 respectively. Genotype results using real time-PCR are shown in Figure 1a. To test the specificity of these assays, selected amplicons of different melting patterns were re-sequenced using an automated DNA sequencer (Beckman Coulter CEQ 2000XL, data not shown).

### Statistical Analysis

To compare allele and genotype frequencies between patients, control and super-control groups, we performed conventional chi-square tests with Yates correction or Fisher exact test using Statcalc (EpiInfo 5.1, Center for Disease control, Atlanta, GA). For statistical analysis of genotype distribution, test for deviation of Hardy-Weinberg equilibrium or two-point association studies, we employed six different tests adapted from Sasieni (deviation from Hardy-Weinberg equilibrium, allele frequency differences test, heterozygous test, homozygous test, allele positivity test and Armitage's trend test) [11]. These calculations were performed in the online resource at the Institute for Human Genetics, Munich, Germany . Significant thresholds for statistical studies were fixed at p < 0.05.

## Results

Looking for somatic mutations within the *NRIP1 *gene, we determined the complete coding sequence of the candidate gene in 20 selected and unrelated somatic endometriotic tissues using bi-directional automated capillary DNA sequencing. In our primary sequencing project we finished 80,600 bp of DNA. Using our methodology, we identified six single nucleotide DNA variants within the coding sequence of the *NRIP1 *gene in various unrelated somatic DNA samples. Two of these variants have been previously identified and they are included in the Single Nucleotide Polymorphism Database (dbSNP) at the National Centre for Biotechnology Information (NCBI)  (Table 3). Five of these mutations alter the amino acid coding sequence of RIP140 protein generating missense mutations (Fig. 1 and Table 3). Although all mutations were detected in somatic DNA, direct molecular analyses of the corresponding blood samples of mutated tissue also contain the same DNA change. This last result implies that the genetic variants identified are germ-line and, consequently, somatic mutations at *NRIP1 *locus are not commonly involved in the pathogenesis of human endometriosis.

**Table 3:** Summary of DNA variants observed within the coding sequence of the *NRIP1 *gene.

DNA variant*	**Amino acid Substitution (change in codon)**	dbSNP** accession number	**Detection in Endometriotic tissue samples (40 chromosomes)**	**Detection in germline DNA derived from endometriosis patients (118 chromosomes)**	**Detection in controls (282 chromosomes)**	**Status**
Nt c.512 G->A	None [Gly75] (ggg to gga)	rs2229741	15/40	57/118	129/282	Common polymorphism
Nt c.949 A->G	His221Arg (cat to cgt)	-	1/40	1/118	3/282	Common polymorphism
Nt c.1608 A->G	Ile441Val (ata to gta)	-	0/20	0/118	4/282	Common polymorphism
Nt c.1629 C->G	Arg448Gly (cga to gga)	rs2229742	9/40	16/118	19/282	Common polymorphism
Nt c.2695 C->T	Ser803Leu (tcg to ttg)	-	2/40	3/118	10/282	Common polymorphism
Nt c.3522 G->T	Val1079Phe (gtt to ttt)	-	1/40	1/118	0/282	Rare Variant/Mutation

*In accordance with genbank number NM_003489.

** dbSNP: the Single Nucleotide Polymorphism Database at the National Centre for Biotechnology Information (NCBI)

To evaluate the polygenic role of *NRIP1 *gene variants in human endometriosis, we decided to preliminary explore the allelic frequencies and genotypes of these mutations in women affected by endometriosis and unselected controls. To conduct genetic association studies, we developed real-time PCR detection protocols using FRET probes for each DNA mutation identified at *NRIP1 *locus. Using these techniques, we genotyped the mutations in 200 unrelated women (59 endometriosis patients, 94 unselected controls and 47 super-control women). Overall, 400 different chromosomes have been scored for each DNA variant (Table 4).

**Table 4:** Association studies of common DNA variants of the *NRIP1 *gene in relation to human endometriosis.

***NRIP1 *polymorphism (change in codon)**	**Genotypes**	**Patients (n = 59)**	**Unselected Controls (n = 94)**	**Super Controls (n = 47)**	**All Controls (n = 141)**	Statistical Analysis*
Gly75Gly (ggg to gga)	aa	15	19	9	28	P = 0.34 (Heterozygous test)
	ag	27	46	27	73	
	gg	17	28	11	39	
Arg448Gly (cga to gga)	cc	44	83	40	123	P = 0.027 (Allele positivity test)
	cg	14	10	7	17	
	gg	1	1	0	1	
Ser803Leu (tcg to ttg)	cc	56	85	46	130	P = 0.59 (Armitage's trend test)
	ct	3	9	1	10	
	tt	0	0	0	0	

*Compares patients versus merged controls. Best p value employing tests for genetic association according to Sasieni (1997). Ile441Val and His221Arg are not analyzed due to small or null sample size in endometriosis samples.

By analyzing the allelic frequencies of DNA variants detected in Spanish population, we classified these variants as common polymorphisms if observed in >1% of chromosomes in controls (*Gly75Gly*, *His221Arg, Ile441Val*, *Arg448Gly *and *Ser803Leu*) or rare variants if observed with a frequency <1% of chromosomes in controls (Table 3). In contrast, *Val1079Phe *allele appears only in a single patient in heterozygous state and none of 141 controls. This data could suggest its involvement in the disease. Reinforcing this hypothesis, Val1079Phe is located close to high-conserved domain of the carboxylic end of RIP140 protein that interacts with retinoic acid nuclear receptor (Fig. 1).

Direct inspection of genotypes in patients revealed three genotype patterns within the *NRIP1 *gene that appear to be over-represented in women affected by endometriosis (p = 0.006, Fisher exact test). The patterns consist of a combination of *Arg448Gly *together with *His221Arg *or *Val1079Phe *variants. We identified three unrelated women affected by endometriosis carrying double heterozygotes (*His221Arg/Arg448Gly *and *Val1079Phe/Arg448Gly*) or homozygote (*Arg448Gly/Arg448Gly*) genotypes for these alleles, respectively. The homozygote (*Arg448Gly/Arg448Gly*) genotype pattern appeared only in 1 of 94 unselected controls (p = 0.016 Fisher exact test) and none of 47 super-control women (p = 0.023, Fisher Exact test), whereas double heterozygotes genotypes did not appear in any control individual. These results suggest that specific combinations of amino acid changes at *NRIP1 *locus could be related to endometriosis etiology with a 99.4% of reliability, although given the scarce sample size the presence of polygenes within *NRIP1 *locus must be proven with a larger and independent re-analysis.

Finally, given the preliminary results, we conducted an small case-control study analyzing all common variants detected within the *NRIP1 *locus (*Gy75Gly*, *Arg448Gly *and *Ser803Leu*). Table 4 shows the results for those test that maximize the differences between case and control groups for each polymorphism. Genotypic distributions of polymorphisms analyzed are in accordance with the Hardy-Weinberg equilibrium law (p > 0.15), indicating no bias due to technical or stratification problems nor evolution-dependent genetic sweep/selection events (data not shown). Interestingly, our analysis revealed that *Arg448Gly *polymorphism appears to be weakly associated with endometriosis in our population (Odds ratio = 2.327, p_allele positivity test _= 0.027). In contrast, no significant association could be achieved when comparing unselected versus super-control women, supporting the accuracy of the selected control panel (p > 0.34 for *Gly75Gly*, p > 0.41 for *Arg448Gly*, and p > 0.1 for *Ser804Leu*).

Overall, our results might support the role of *NRIP1 *gene in endometriosis, although given the small sample size, we propose an extensive re-analysis by increasing the sample size to confirm our results.

## Discussion

Endometriosis is a complex disease affecting 10–15% of women at reproductive age. Very few genes are known to be altered in this pathology. Molecular genetic analyses provide some evidence of genetic association in case-control studies analyzing *Estrogen Receptor 1 *(*ESR1 *OMIM 133430) and *Cytochrome P450*, *Family 19*, *Subfamily A*, *Polypeptide 1 *(*CYP19 *OMIM 107910) genes. Interestingly, both loci are involved in oestrogen mechanism of production and action [12,13]. In addition, other nuclear receptor genes, such as *Progesterone Receptor *(*PGR *OMIM 607311) and *Peroxisome Proliferative Activated Receptor*, *Gamma *(*PPARG *OMIM 601487) gene have been associated with endometriosis in other case-control studies [14,15]. The involvement in endometriosis of loci related to detoxification has been also studied and replicated [16-18].

Given these preliminary findings and the importance of steroid receptors in uterine physiology [19] and endometriosis pathogenesis [1,20], the biochemical pathways involved in steroids production, degradation or mechanisms of action appear to be strong candidates for endometriosis etiology and many other phenotypes related to human fertility.

Following this working hypothesis, targeting disruption of nuclear receptors and their regulators such as *nrip1 *or *CCR4-NOT transcription complex*, *subunit 7 *(*cnot7 *GenBank AK009561) in animal models have provided direct evidence of the importance of nuclear receptor homeostasis in male and female reproduction [3,21-24].

Here we present the first structural analysis of the human *NRIP1 *gene in relation to human disease. It is of interest to mention that the dbSNP includes 26 SNPs for *NRIP1 *gene currently. Eighteen of these variants are located within the 3'untranslated region (3'UTR), this genomic region has not been covered in this study, and the remaining ones are coding SNPs. According to GenBank, only three SNPs in the 3'UTR region and two coding SNPs have been validated in population based studies including more than 150 chromosomes. The rest of the SNPs are the result of the bioinformatic alignment of different cDNA and genomic clones. The allele frequencies here presented for *Gly75Gly *(dbSNP rs2229741) and *Arg448Gly *(dbSNP rs2229742) polymorphisms are very similar to those included in dbSNP (data not shown).

Overall, our results are preliminary providingt suggestive, but not definitive, evidence of *NRIP1 *gene involvement in human endometriosis. We think that conclusive proofs of involvement will be achieved throughout re-analyses of this study in independent cohorts of patients and controls, rather than performing functional analyses of the missense mutations observed. The detection of functionality of DNA variants involved in complex traits such endometriosis, is near to be impossible using conventional technologies because the effect from single genetic variant/mutation is expected to be very small and it is only the joint effect of several susceptibility genes that leads to the disease [25]. In this sense, we are currently recruiting a higher number of patients and controls to perform a proper re-analysis of our results.

Regarding *Arg448Gly *polymorphism, we propose that the variant could act as a low penetrance allele related to human endometriosis. The molecular mechanism of this mutation is not well understood, although its location and degree of conservation provide some interesting clues. In fact, Arg448 residue of RIP140 protein is completely conserved among humans, rats, mice, gallus and xenopus (Fig. 1b). Moreover, the non-conservative substitution detected (*Arg448Gly*) might affect the Carboxyl terminal binding protein (CTBP) interacting motif of RIP140 protein that is located close to this amino acid residue (Fig. 1b).

On the basis of genotype analysis in affected women, we propose that *Arg448Gly *mutation could act in concert with other genetic variants within *NRIP1 *or other loci. In this way, we found a single woman affected by endometriosis simultaneously carrying *Val1079Phe *mutation and *Arg448Gly *polymorphism both in a heterozygous state. *Val1079Phe *also arises in an inter-specific conserved residue. Moreover, this mutation is located close to (and may disrupt) the retinoid acid receptor interacting motif "LTKTNPILYYMLQK" of RIP140 protein (Fig. 1b). Supporting its involvement in the disease, we have not identified the *Val1079Phe *mutation in 282-control chromosomes. Intriguingly, retinoid acid receptors alpha, gamma 2 and its regulator cnot7 have been involved in male sterility [24,26,27]. Moreover, the presence of multiple specific functional rare variants in affected patients have been recently proposed and evaluated [28]. This hypothesis is an alternative to explain the genetic component of complex traits in front of the widely accepted common disease common variant hypothesis [29].

Finally, we identified a single patient carrying a unique genotype combination comprising *His221Arg *rare variant and, again, *Arg448Gly *polymorphism. The absence of inter-specific amino acidic conservation and the inexistence of known functional domains close to *His221Arg *variant do not support the functionality of *His221Arg *allele. However, *His221Arg *only appears combined with *Arg448Gly *in a woman affected by endometriosis. This combination never appears in 141 unrelated controls. Functional assays or large cohorts analyses will help to elucidate its involvement in this pathology.

Our results support that *NRIP1 *gene might contain alleles related to endometriosis in humans. *NRIP1 *gene encodes a highly pleiotropic nuclear receptor co-regulator (RIP140) [30]. This protein interacts and regulates multiple members of the nuclear receptor super-family. Some of them have been associated with endometriosis. The wide repertoire of RIP140 targets might explain the complex pathological findings observed in human endometriosis. In fact, a recent report revealed a complex mechanism, involved in endometriosis and other oestrogen-related traits, by which ER-mediated oestrogen signaling is modulated by a co-regulatory-like function of activated AhR/Arnt dioxin receptor complex, giving rise to adverse oestrogen-related actions of dioxin-type environmental contaminants [31]. Intriguingly, oestrogen and dioxin nuclear receptor pathways are modulated by RIP140 protein [32,33].

According to previous data, mouse models reports and the present results, *NRIP1 *gene appears to be an attractive gene for human endometriosis etiology and other related pathologies. In addition, we have found a genetic interaction of *NRIP1 *gene with Estrogen receptor alpha and beta (*ESR1 *and *ESR2*) genes in two estrogen-dependent diseases such as male infertility [34] and osteoporosis (manuscript in preparation). For these reasons, further evaluation of *NRIP1 *gene in many oestrogen-related phenotypes is warranted.

## Conclusion

Our results suggest that *NRIP1 *gene variants, separately or in combinations, might act as predisposing factors for human endometriosis. According to previous data, mouse models reports and the present results, *NRIP1 *appears to be an attractive candidate gene for human endometriosis etiology and other estrogen-related pathologies.

## Competing interests

The author(s) declare that they have not competing interests.

## Authors' contributions

VC, JAS, MC, MAL, FC and VL carried out the recruitment and classification of patients and controls and the biological samples management. RR and JJG carried out the molecular genetic studies and participate in the analysis and interpretation of data. LMR and AR carried out the design of the study, performed the statistical analyses and the interpretation of data.

All authors have been involved in drafting the article or revising it critically for important intellectual content and have given final approval of the version to be published.
